# A Review of Ionospheric Scintillation Models

**DOI:** 10.1007/s10712-015-9319-1

**Published:** 2015-01-28

**Authors:** S. Priyadarshi

**Affiliations:** Space Research Centre, Bartycka 18a, 00-716 Warsaw, Poland

**Keywords:** Ionosphere, Scintillation, Plasma instability, Ionospheric irregularities, Trans-ionospheric communication

## Abstract

This is a general review of the existing climatological models of ionospheric radio scintillation for high and equatorial latitudes. Trans-ionospheric communication of radio waves from transmitter to user is affected by the ionosphere which is highly variable and dynamic in both time and space. Scintillation is the term given to irregular amplitude and phase fluctuations of the received signals and related to the electron density irregularities in the ionosphere. Key sources of ionospheric irregularities are plasma instabilities; every irregularities model is based on the theory of radio wave propagation in random media. It is important to understand scintillation phenomena and the approach of different theories. Therefore, we have briefly discussed the theories that are used to interpret ionospheric scintillation data. The global morphology of ionospheric scintillation is also discussed briefly. The most important (in our opinion) analytical and physical models of scintillation are reviewed here.

## Introduction

The radio scintillation phenomenon is very similar to the twinkling of the stars in the visible part of the electromagnetic spectrum which are due to variations in tropospheric density due to turbulence. Scintillation is a fact of life for a number of radio communication and navigation systems that have to operate through the auroral or equatorial ionosphere (Crane 1977). Space-diversity and time-diversity coding schemes are required to mitigate the effects of scintillation. Random fluctuations in the refractive index of the medium cause fluctuations of a very high-frequency radio signal passing through the medium, and this effect is referred as scintillation. In particular, scintillations are fluctuations of the parameters of trans-ionospheric waves, i.e., their phase, amplitude, direction of propagation and polarization.

**Fig. 1:**
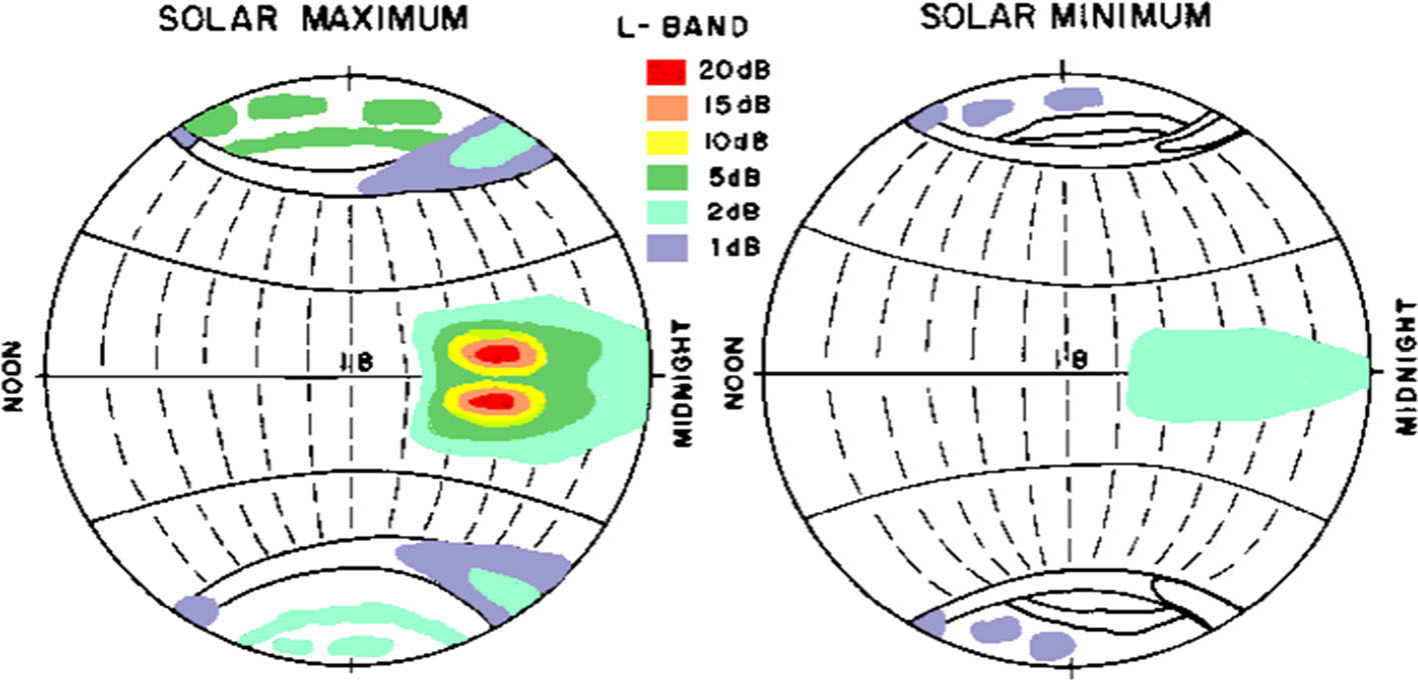
Global variation of amplitude scintillation fades at L band (after Basu et al. 1988a, b, colored by A.W. Wernik)

Scintillation observations have been used to identify and diagnose irregular structure in highly varied propagation media. Research fields like atmospheric physics, geophysics, ionospheric physics, ocean acoustics, astronomy and radio physics have benefitted through scintillation research (Rino 2011). In the past, voluminous studies based on observations have been performed on ionospheric amplitude and phase scintillation. The global distribution of ionospheric amplitude scintillation is shown in Fig. 1. The major scintillation activity is observed during the solar maximum period, near the magnetic equator and in the midnight sector (Basu et al. 1988a, b).

The theory of radio wave propagation in random media has been developed, allowing for the physical interpretation of scintillation data in terms of the properties of the ionosphere’s irregular structure (Tatarski 1971; Yeh and Liu 1982; Bhattacharyya et al. 1992). It has been found that the irregularities producing scintillations are predominantly localized in the F layer, near the peak of plasma density, at ~ 300 km altitude. To explain the generation of plasma irregularities, various kinds of plasma instabilities were considered. It seems that the consensus that has been reached is that the generalized Rayleigh–Taylor instability is the primary mechanism generating equatorial plasma density irregularities of intermediate scale length, i.e., with sizes of the order of a few hundreds of meters to several kilometers (Kelley 1989). Plasma in the high-latitude F-region ionosphere is highly structured on a scale from tens of meters to tens of kilometers (Dyson et al. 1974; Clark and Raitt 1976; Phelps and Sagalyn 1976; Tsunoda 1988).

In this paper, we will present and discuss the most important models of ionospheric scintillation. Following Maria (1997), a model is a representation of the construction and operation of a certain system. In our case, as a system, we identify the propagation of radio waves through the ionosphere with fluctuating electron density. The model is validated using simulations under a known input and comparing the model and system outputs (Maria 1997; Winsberg 2013). In the case of scintillation modeling, the simulation is a solution of the equations modeling the wave propagation through the irregular ionosphere. The present paper discusses briefly scintillation mechanisms, different scintillation theories and the morphology of high- and low-latitude scintillations. As the main objective of the paper, we will review some existing scintillation models.

## Background

### Scintillation Phenomenon

Much of our knowledge of ionospheric scintillation and the irregularities that cause the scintillation has been derived from the many observations taken over the past six decades. The scintillation of radio signals is a consequence of the presence of random fluctuations of the refractive index related to the electron density irregularities. These fluctuations distort the original wavefront of the signal. Consequently, they give rise to a randomly phase-modulated wave. Formally, ionospheric scintillation can be defined as a random modulation imparted to propagating wave fields by structure in the propagation medium (Rino 2011). As the satellite, and/or ionosphere, or both, moves relative to the receiver, temporal variations of intensity and phase are recorded (Wernik et al. 2003).

### Scintillation Theory

Different theories are used to interpret ionospheric scintillation data. Since the scintillation phenomenon is being studied by direct measurements from a number of satellites using multi-frequency phase coherent beacon signals, we will focus on the full structure of the complex signal. From the beginning of the advent of ionospheric scintillation phenomenon, a comprehensive and wide literature exists; the January 1975 issue of RadioScience was devoted to this subject. Weak-scatter theory, the Rytov approximation, single, thin or multiple phase screen, multiple-scatter theory are the theories used to study scintillation data for last six decades. We shall discuss each one of them very briefly.

Under the assumption that the characteristic scale size and characteristic scale of the temporal variations of the refractive index fluctuations are much larger than the respective radio wavelength and wave period, the vector wave equation can be replaced with the scalar wave equation

1 $$\nabla^{2} E + k^{2} [1 + \varepsilon_{1} ({\mathbf{r}},t)]E = 0$$

where *ε*_1_(**r**,*t*) is the fluctuating part of the dielectric permittivity caused by electron density irregularities, and *k* ^2^ = *k*_0_^2^ 〈ε〉 with *k*_0_—the wave number in free space, 〈ε〉—the average dielectric permittivity; (**r**,*t*) specifies the location of the irregularity is space and time.

Equation (1) is a differential equation with randomly fluctuating coefficients which forms the basis of the scintillation theory. To solve the equation, one has to resort to certain approximations. We assume that a plane wave is incident normally on a plane-parallel irregular slab. If the incident direction is identified with the *z* direction of the coordinate system, *u*(***r*** *,t*) is the complex amplitude of the radio wave so that

2 $$E({\mathbf{r}},t) = u({\mathbf{r}},t)\exp ( - ikz)$$

the substitution of (2) into (1) leads to the following equation

3 $$- 2ik\frac{\partial u}{\partial z} + \nabla^{2} u = - k^{2} \varepsilon_{1} ({\mathbf{r}},t)u$$

If the wavelength of the radio signal *λ* is much smaller than the characteristic scale *l*_0_ of the irregularities, then 2 *k*|∂*u*/∂*z*| ≫ |∂^2^ *u*/∂*z* ^2^| and (3) reduces to the following parabolic equation

4 $$- 2ik\frac{\partial u}{\partial z} + \nabla_{ \bot }^{2} u = - k^{2} \varepsilon_{1} ({\mathbf{r}},t)u$$

where ∇_⊥_^2^ is the transverse Laplacian.

The Laplacian term represents the diffraction of the wave, while the right-hand side of (4) represents random phase shifts caused by the refractive index fluctuations. If *k*/*L* ≫ 1/*r*_0_^2^, where *r*_0_ is the outer scale of the irregularities and *L* is the irregular slab thickness, the Laplacian term can be neglected as compared to the gradient term and (4) reduces to

5 $$\frac{\partial u}{\partial z} = - i\frac{k}{2}\varepsilon_{1} ({\mathbf{r}},t)u$$

The condition *k*/*L* ≫ 1/*r*_0_^2^ is equivalent to the requirement that the size of the first Fresnel zone $\sqrt {\lambda L}$ is much less than the outer scale *r*_0_. Under this condition, diffraction is ignored and the complex amplitude is equal to

6 $$u({\mathbf{r}},t) = A_{0} \exp [ - i\phi ({\mathbf{r}},t)]$$

where the wave phase *φ*(***r*** *,t*) = *φ*(***ρ*** *,z,t*) is given by

7 $$\phi (\rho ,z,t) = \frac{k}{2}\int\limits_{0}^{z} {\varepsilon_{1} (\rho ,z^{{\prime }} ,t){\text{d}}z^{{\prime }} = - \lambda r_{e} \Delta N_{T} (\rho ,z,t)}$$

In (7), *r*_*e*_ is the classical electron radius, Δ*N*_*T*_ is the fluctuation of the electron content between transmitter and receiver on the ground, and ***ρ*** is a vector in a plane transverse to the propagation direction.

Equations (6) and (7) describe so-called phase screen approximation or model. The concept of the phase-changing screen was introduced by Booker et al. (1950) and Ratcliffe (1956) and later advanced and developed by Rino (1976). Below the screen, the wave propagates in free space, undergoing phase mixing. To express mathematically this effect, we set the right-hand side of (4) equal to zero:

8 $$- 2ik\frac{\partial u}{\partial z} + \nabla_{ \bot }^{2} u = 0$$

The solution of this equation with the “initial” condition as given by (6) can be expressed in the form of the Fresnel diffraction formula:

9 $$u({\varvec{\uprho}},z,t) = \frac{{ikA_{0} }}{2\pi z}\int\limits_{ - \infty }^{\infty } {\int\limits_{ - \infty }^{\infty } {\exp \left\{ { - i\left[ {\phi ({\varvec{\uprho}}^{{\prime }} ,z,t) + \frac{k}{2z}|{\varvec{\uprho}} - {\varvec{\uprho}}^{{\prime }} |^{2} } \right]} \right\}{\text{d}}{\varvec{\uprho}}^{{\prime }} } }$$

**Fig. 2:**
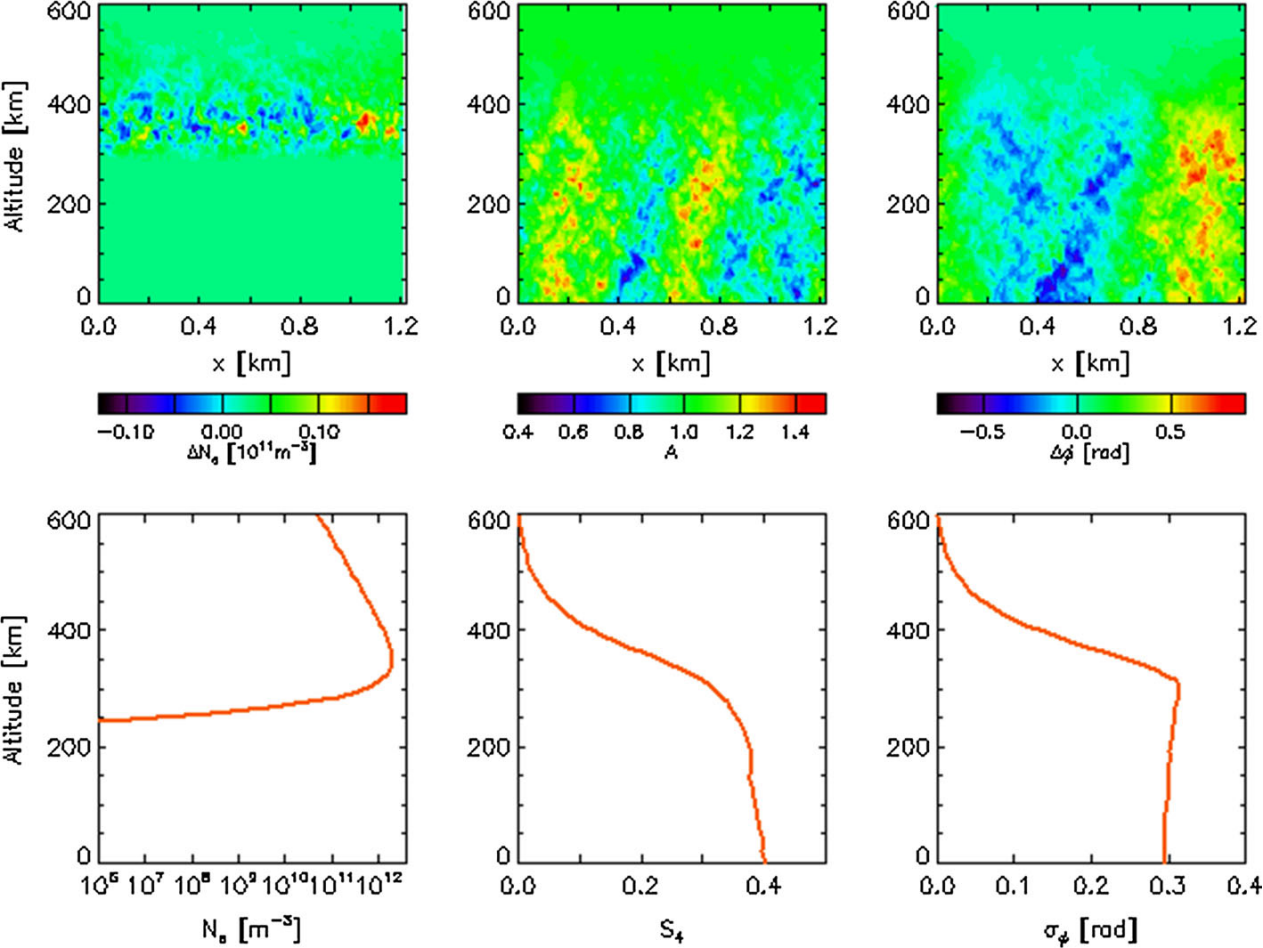
Variations in the vertical plane of simulated electron density fluctuations (*upper left panel*), signal amplitude (*upper middle panel*) and phase (*upper right panel*). The electron density height profile is shown in the *lower left panel*, and the scintillation index *S*_4_ and phase variance *σ*_*ϕ*_ profiles in the *lower middle* and *right panels*, respectively

The approach in which scintillation is considered as propagation inside the irregular medium with the succeeding propagation in free space down to the receiver is called split-step algorithm or model. If the wave propagates in a non-uniform ionosphere, in which the background electron density varies with the altitude, then one can use the split-step algorithm several times along the propagation path, adjusting the background density to the required profile and using the previous step results as the initial condition for the next step. An example of the simulated scintillation in the L2 band is shown in Fig. 2. It was assumed that the electron density profile is a Chapman function with the peak density 2 × 10^12^ electrons/m^3^ at the peak height 350 km. The irregularities are characterized by the power law spectrum with the 3D spectral index equal to 3.5. One can see that the main contribution to scintillation is due to those irregularities which are close to the peak electron density.

Scintillation is a stochastic (random) phenomenon, and to validate scintillation theories, one should compare observed and theoretical statistics. The statistics most easily calculated from measurements are the scintillation index *S*_4_ (normalized standard deviation of the signal power/intensity) and the phase standard deviation *σ*_*ϕ*_. The derivation of the theoretical statistics requires the calculation of the moments of the complex amplitude *u*(***r*** *,t*) and is difficult. It requires an assumption about the probability distribution of the random electron density fluctuations. Usually, it is assumed that this is a Gaussian with zero mean. If so, then the first moment of the complex amplitude 〈*u*〉 is given by Yeh and Liu (1982) as:

10 $$\left\langle u \right\rangle = \exp ( - \varphi_{0}^{2} /2)$$

where *ϕ*_0_^2^ is the variance of the phase introduced by the medium.

The average signal intensity is equal to

11 $$\left\langle {uu^{*} } \right\rangle = A_{0}^{2}$$

which does not change as the wave propagates through the medium. This reflects the conservation of wave energy.

Derivation of the higher order moments of *u*(***r*** *,t*) is cumbersome and will not be given here. We refer interested readers to the review by Yeh and Liu (1982) where further references can be found.

Formally, the phase screen approach, as described by (6) and (9), is not limited to a weak scintillation. However, often—especially at higher frequencies—*S*_4_ and *σ*_*ϕ*_ can be approximated assuming that the phase variance *ϕ*_0_^2^ on a screen is much smaller than one. This assumption is known as the shallow screen approximation. In the shallow screen approximation, the statistics of a signal can be related to the statistics of the phase fluctuations on a screen and therefore to the statistics of the scattering medium.

Let us define the logarithmic amplitude *χ* and departure phase *S*_1_ by the following relation:

12 $$u({\varvec{\uprho}},z,t) = A_{0} \exp [\chi ({\varvec{\uprho}},z,t) - iS_{1} ({\varvec{\uprho}},z,t)] = A_{0} \exp [i\psi ({\varvec{\uprho}},z,t)]$$

For a shallow screen, the power spectra of *χ* and *S*_1_ are given by Yeh and Liu (1982) as:

13 $$\begin{aligned} F_{\chi } ({\varvec{\upkappa}}_{ \bot } ) = & \sin^{2} ({\varvec{\upkappa}}_{ \bot }^{2} z/2k)F_{\phi } ({\varvec{\upkappa}}_{ \bot } ) = 2\pi \lambda^{2} r_{e}^{2} \sin^{2} ({\varvec{\upkappa}}_{ \bot }^{2} z/2k)F_{N} ({\varvec{\upkappa}}_{ \bot } ,0) \\ F_{S} ({\varvec{\upkappa}}_{ \bot } ) = & \cos^{2} ({\varvec{\upkappa}}_{ \bot }^{2} z/2k)F_{\phi } ({\varvec{\upkappa}}_{ \bot } ) = 2\pi \lambda^{2} r_{e}^{2} \cos^{2} ({\varvec{\upkappa}}_{ \bot }^{2} z/2k)F_{N} ({\varvec{\upkappa}}_{ \bot } ,0) \\ \end{aligned}$$

where **κ**_⊥_ is the transverse spectral wave vector, and *F*_*φ*_ and *F*_*N*_ are power spectra of phase and density, respectively.

#### The Weak-Scatter Theory

Booker et al. (1950) first introduced the weak-scatter theory. The work of Ratcliffe (1956), Bowhill (1961), Briggs and Parkin (1963) and Budden (1965a, b) improved this theory later. This theory resembles the first Born approximation solution to the vector wave equation. Booker et al. (1950) and Ratcliffe (1956) introduced the concept of the phase-changing screen. The weak-scatter theory is almost always formulated within the framework of a weak phase-changing screen. At the edge of the phase-changing screen, *z*_0_, the scalar wave field *u*(**ρ**,*z*_0)_ is expressed as follows

14 $$u({\varvec{\uprho}},z_{0} ) = \exp \{ i\phi ({\varvec{\uprho}};z_{0} )\}$$

where *ϕ*(ρ,*z*_0_) is obtained by a straight-line path integration (Rino 1976). The variance of *ϕ*(ρ,*z*_0_) is denoted by *ϕ*_0_^2^; in weak-scatter theory, the variance of *ϕ* (ρ,*z*_0_) is numerically equal to σ^2^ *L*, where σ^2^ is a linear scattering coefficient and *L* is the layer thickness. For an ionized medium, the variance can be expressed as follows

15 $$\sigma^{2} L = \phi_{0}^{2} = r_{e}^{2} \lambda^{2} L\sec^{2} \theta \iint {\varPhi_{{\Delta N_{e} }} \left( {k, - \tan \theta \hat{a}_{{k_{T} }} \cdot k} \right)\frac{{{\text{d}}k}}{{(2\pi )^{2} }}}$$

where θ is the incidence angle, κ = (2π/λ)â_κ_ is the wave vector, âk_Τ_ is the projection of the wave vector onto the x–y plane, and Φ_ΔNe_ (κ,κ_z_) is the three-dimensional spectral density function for the electron density irregularity. This equation shows that *ϕ*_0_^2^ ∝ *L* sec^2^ θ for an isotropic medium. The wave field can be described to statistics of the second order by the mutual coherence function

16 $$R_{u} (\Delta {\varvec{\uprho}};z) \triangleq \left\langle {u({\varvec{\uprho}},z)u^{*} ({\varvec{\uprho}}^{{\prime }} ;z)} \right\rangle - \left| {\left\langle {u({\varvec{\uprho}},z)} \right\rangle } \right|^{2}$$

and the complementary function is expressed as follows

17 $$B_{u} (\Delta {\varvec{\uprho}};z) = \left\langle {u({\varvec{\uprho}},z)u({\varvec{\uprho}}^{{\prime }} ,z)} \right\rangle - \left\langle {u({\varvec{\uprho}},z)} \right\rangle^{2} .$$

Only the intensity (*I* ≈ |*u*|^2^) fluctuations are usually measured, the principal observable being

18 $$R_{I} (\Delta {\varvec{\uprho}};z) \triangleq \frac{{\left\langle {II^{{\prime }} } \right\rangle - \left\langle I \right\rangle \left\langle {I^{{\prime }} } \right\rangle }}{{\left\langle I \right\rangle \left\langle {I^{{\prime }} } \right\rangle }}$$

The commonly used amplitude scintillation index *S*_4_ is simply expressed as [*R*_*I*_(0;*z*)]^1/2^.

#### The Rytov Approximation

The weak-scatter theory is equivalent to the first Born approximation, which takes the form of a scalar wave field u (ρ,*z*) ≈ 1 + ψ. Thus, we must have |ψ| ≪ 1. By comparison, the Rytov approximation takes the form u (ρ,*z*) ≈ exp{ψ} with the same perturbation function ψ (Rino 1976). It follows that all the previous weak-scatter results apply to log-amplitude and phase. It was originally thought that the Rytov solution had a greater range of validity than the Born solution (e.g., Barabanenkov et al. 1971), but this has since been shown to be not strictly correct. The amplitude fluctuation must still be small, but not the phase.

#### The Gaussian Phase Screen

In the Gaussian phase screen model, *ϕ* (ρ, *z*_0_) (where *z*_0_ is edge of the phase-changing screen) is assumed to be a Gaussian random process. The power of this model lies in the fact that all observables are calculable (Rino 1976). For example, one can easily show that

19 $$R_{u} (\Delta {\varvec{\uprho}};z_{0} ) = \exp \{ - \phi_{0}^{2} (1 - {\varvec{\uprho}}_{{\Delta N_{e} }} (\Delta {\varvec{\uprho}}))\} - \exp \{ - \phi_{0}^{2} \}$$

and

20 $$B_{u} (\Delta {\varvec{\uprho}};z_{0} ) = \exp \{ - \phi_{0}^{2} (1 + {\varvec{\uprho}}_{{\Delta N_{e} }} (\Delta {\varvec{\uprho}}))\} - \exp \{ - \phi_{0}^{2} \}$$

where *R*_*u*_ is the mutual coherence function, *B*_*u*_ is complementary function, ρ_Δ*Ne*_ (Δρ) is the two-dimensional (in a plane perpendicular to the direction of propagation) correlation function of the electron density fluctuations, and the used result is 〈*u*〉 = exp{−½*ϕ*_0_^2^}, valid if *ϕ* (ρ, *z*_0_) is a Gaussian field.

#### Multiple-Scatter Theory

The parabolic approximation to the scalar wave equation can be used to derive a system of differential equations for the moments of the scalar wave field u. In deriving these equations, one makes use of the so-called Markov approximation meaning that, under the forward scattering assumption, the field at any height *z*′ depends only on those irregularities in the region *z* < *z*′. Validity conditions have been derived by Tatarski (1971) for the general case and by Beran (1970) and others for the mutual coherence function equation. The validity condition takes the form σ^2^Δ*z* ≪ 1, where σ^2^ is a linear scattering coefficient. Tatarski (1971) gives Δ*z* ~ λ, whereas Beran (1970) gives a much more restrictive condition. The intensity correlation function has been numerically evaluated by Yeh et al. (1975). Approximate formulas for the two-frequency correlation functions have been given by Liu et al. (1974).

A power law spectrum of the form 1/κ^p^ for all values of κ has several difficulties (Yeh and Liu 1977). For example, for a spectral index *p* > 2, its associated correlation function will not exist. Also for any finite value of *p,* the spectral moments will fail to exist above a certain order. To avoid this situation, a finite outer scale was introduced by Tatarski (1971). The theory discussed here so far assumes, at least implicitly, that the outer scale is finite, whereas the ionosphere does not show a well-defined outer scale cutoff (Crane 1976).

Rino (1976) shows observations of a navy navigation satellite signal at 150 MHz which justify the omnipresence of large-scale structures that induce trend-like but, nonetheless, random phase fluctuations well in excess of 1 rad. The weak-scatter theory cannot properly interpret such signal structures. The Rytov approximation used by Crane (1976) is appealing here since it is valid for large phase perturbations associated with small-amplitude fluctuations. Here, it would be best to use multiple-scatter theory. Primarily, phase perturbations are associated with large-scale structures, while diffraction gives rise to a Gaussian component of *R*_*u*_ (mutual coherence function), *B*_*u*_ (complementary function) and finally to the electron density function.

### Global Morphology Ionospheric Scintillation

Starting with post-World War II studies of the fading of radio star sources and continuing with the fading of signals from the Sputnik satellite, a gigantic amount of data has been acquired to study the effect of ionospheric irregularities on signals propagating through the ionosphere. The invited review paper by Aarons (1982) reviewed attempts to organize the amplitude and phase scintillation data into equatorial-, middle- and high-latitude morphologies. Globally, there are three major regions of scintillation activity. The equatorial region comprises an area within ±20° of latitude of the magnetic equator. The high-latitude region, for the purposes of the scintillation description, comprises the area from the high-latitude edge of the trapped charged particle boundary (Van Allen outer belt) into the polar region. All other regions are termed as “middle latitudes.” In all regions, there is a pronounced nighttime maximum of scintillation activity. In the equatorial regions, the activity begins only after sunset. Even in the polar region, there appears to be a greater scintillation occurrence during the dark months than during the months of continuous Sun. At the equator, the Earth’s magnetic field is parallel to the Earth’s surface and is oriented magnetic N–S, while at the pole it is vertical. This strong characterization in relation to the geomagnetic field configuration makes these regions the most affected by the formation of the ionospheric irregularities which are potentially dangerous for communication and navigation radio systems. Both ground-based scintillation measurements and in situ satellite data show that ionospheric irregularities are concentrated near the magnetic equator, where they are observed in the pre-midnight period, in auroral zone during the nighttime period, and in the polar region at all local times (Wernik et al. 2004).

Equatorial scintillation-producing ionospheric irregularities are described using the **E** × **B** mechanism and/or the Rayleigh–Taylor mechanism, in which a large (scale of about 100 km) volume of depleted ionization is driven through the F region (Scannapieco and Ossakow 1976). The depleted volume leaves a trail, or plume, of small-scale (tens of centimeters to a meter) irregularities surrounding the depletion, which can extend well through the F-layer peak.

The most intense F-region irregularities in the high-latitude ionosphere seem to be produced by convective plasma processes and, in particular, by the fluid **E** × **B** (gradient-drift) interchange instability. Irregularities are produced by convectively mixing plasma across a mean plasma density gradient. The transport of higher-density plasma into regions of lower-density plasma (and vice versa) leads to the development of an irregularity spectrum that extends in scale from about 10 km down to the ion gyroradius (Tsunoda 1988). Irregularities with this range of scales are not independent from larger-scale plasma structures, that are produced by other means, to those of smaller-scale irregularities.

Scintillation activity at middle latitudes is not as intense as that encountered at equatorial, auroral or polar latitudes. The problem with describing scintillation activity at midlatitudes is that at times it is an extension of phenomenon at equatorial and auroral latitudes. Scintillation activity in 1979–1981, years of high sunspot number, was observed to be high at Hawaii, Japan (Aarons 1982). These effects were possibly caused by equatorial phenomena during years of high sunspot number. The depletion regions that originate at equatorial latitudes do then move to higher altitudes. These irregularities should maintain an altitude >2,000 km. The perturbing effects of these regions and the higher electron densities during high sunspot number years might combine to provide effects along the lines of force of the geomagnetic field, thus extending equatorial activity to the “lower” middle latitudes. At high latitudes, the irregularity boundary moves equatorward during years of high sunspot number and during magnetic storms. Auroras have been observed in the southern USA, along the 70°W meridian. Scintillation activity is present at these times at these lower latitudes when and where optical aurora is seen.

Another complicating factor in midlatitude scintillation morphology is the effect of sporadic E. Several studies in the past few decades have shown that intense sporadic E yields scintillation. The behavior of sporadic E is totally different from the morphology of F-layer irregularities. Thus, two independent variables produce the fading phenomena. At middle latitudes, there is a high occurrence of daytime sporadic E resulting in a second maximum of scintillation. Nighttime sporadic E adds to the effects of F-layer irregularities.

It is important to understand the global morphology of ionospheric scintillation since it will help users to differentiate between fluctuations produced by ionospheric irregularities and those of equipmental or man-made origin.

## Scintillation Models

We have divided scintillation models into three groups—analytical models, global climatological models and models based on in situ data.

### Analytical Models

We shall here consider five such models produced by different research groups.

#### Model of Fremouw and Rino (1973)

Fremouw and Rino (1973) presented the first analytical model of scintillations. The model was suitable for estimating the rms fluctuation in the received signal strength (i.e., the scintillation index) to be expected on a given trans-ionospheric VHF/UHF (but not SHF) communication link, under average scintillation conditions. By average scintillation conditions is meant those conditions to be expected on the average for a given geomagnetic latitude, time of day, day of the year, and sunspot number. Thus, the model does not address the question of variations in scintillation index from its mean value for a given set of the above independent variables. They assumed the center height of the irregular layer to be 350 km, the thickness of the irregular layer 100 km, the ratio of the scale size along the geomagnetic field to that transverse to it of 10, the transverse scale size (defined as a distance over which the spatial correlation falls to 1/e of its maximum value) = ξ_0_ (transverse irregularity scale size) and the rms fluctuations of electron density = Δ*N*. The model for Δ*N* consists of four additive terms, the influence of each being dominant in different regimes of geomagnetic latitude, as follows

21 $$\Delta N = \Delta N_{\text{eq}} \left( {R,D,t,\lambda } \right) + \Delta N_{\text{mid}} \left( {t,\lambda } \right) + \Delta N_{\text{hi}} \left( {R,t,\lambda } \right) + \Delta N_{\text{aur}} \left( {R,t,\lambda } \right)$$

where the subscripts are eq(equatorial region), mid(midlatitude region), hi(high-latitude region) and aur(auroral region) and expressions for these different regions are as given below:

22 $$\begin{aligned} \Delta N_{\text{eq}} = &\,(5.5 \times 10^{9} )(1 + 0.05R) \\ & \cdot \left[ {1 - 0.4\cos \pi \left( {\frac{D + 10}{91.25}} \right)} \right] \\ & \cdot \left\{ {\exp \left[ { - \left( \frac{t}{4} \right)^{2} } \right] + \exp \left[ { - \left( {\frac{t + 23.5}{3.5}} \right)^{2} } \right]} \right\} \\ & \cdot \left\{ {\exp \left[ { - \left( {\frac{\lambda }{12}} \right)^{2} } \right]} \right\}e1/{\text{m}}^{3} \\ \end{aligned}$$

23 $$\begin{aligned} \Delta N_{ \hbox{min} } = & (6.0 \times 10^{8} )\left( {1 + 0.4\cos \frac{\pi t}{12}} \right) \\ & \cdot \left\{ {\exp \left[ { - \left( {\frac{\lambda - 32.5}{10}} \right)^{2} } \right]} \right\}e1/{\text{m}}^{ - 3} \\ \end{aligned}$$

24 $$\Delta N_{\text{hi}} = (2.7 \times 10^{9} )\left\{ {1 + {\text{erf}}\left[ {\frac{\lambda - \lambda (R,t)}{{0.02\lambda_{\text{b}} (R,t)}}} \right]} \right\}e1/{\text{m}}^{ - 3}$$

25 $$\begin{aligned} \Delta N_{\text{aur}} = & (5.0 \times 10^{7} )R \\ & \cdot \left\{ {\exp \left[ { - \left( {\frac{\lambda - 70 + 2\cos (\pi t/12)}{0.03R}} \right)^{2} } \right]} \right\}e1/{\text{m}}^{ - 3} \\ \end{aligned}$$

26 $$\lambda_{\text{b}} = 79 - 0.13R - (5 + 0.04R) \cdot \cos (\pi t/12)\,{\text{degrees}}$$

where *R* is the sunspot number, *D* is the day of the year, *t* the local time of the day in hours, and λ the geomagnetic latitude in degrees. These equations show the linear dependence of the scintillation on *R*, *D*, *t* and λ (geomagnetic latitude in degrees).

Using the assumed Δ*N*, Δ*h*, ξ_0_ and irregularity axial ratio a, the rms phase at the exit from the irregular layer can be calculated using

27 $$\phi_{ 0} = \pi^{1/4} r_{e} \lambda \left[ {(a\xi_{0} \sec i)^{1/2} /\beta^{1/2} } \right](\Delta h)^{1/2} (\Delta N)$$

where *r*_*e*_ is the classical electron radius, λ the wavelength of the wave, i the incidence angle of the radio wave on the irregular layer, Δ*h* the thickness of the irregular layer, ξ_0_ the transverse irregularity scale size, and β = *a* ^2^ sin^2^ω + *b* ^2^ cos^2^ω, where ω is the angle between the magnetic field and the ray path.

**Fig. 3:**
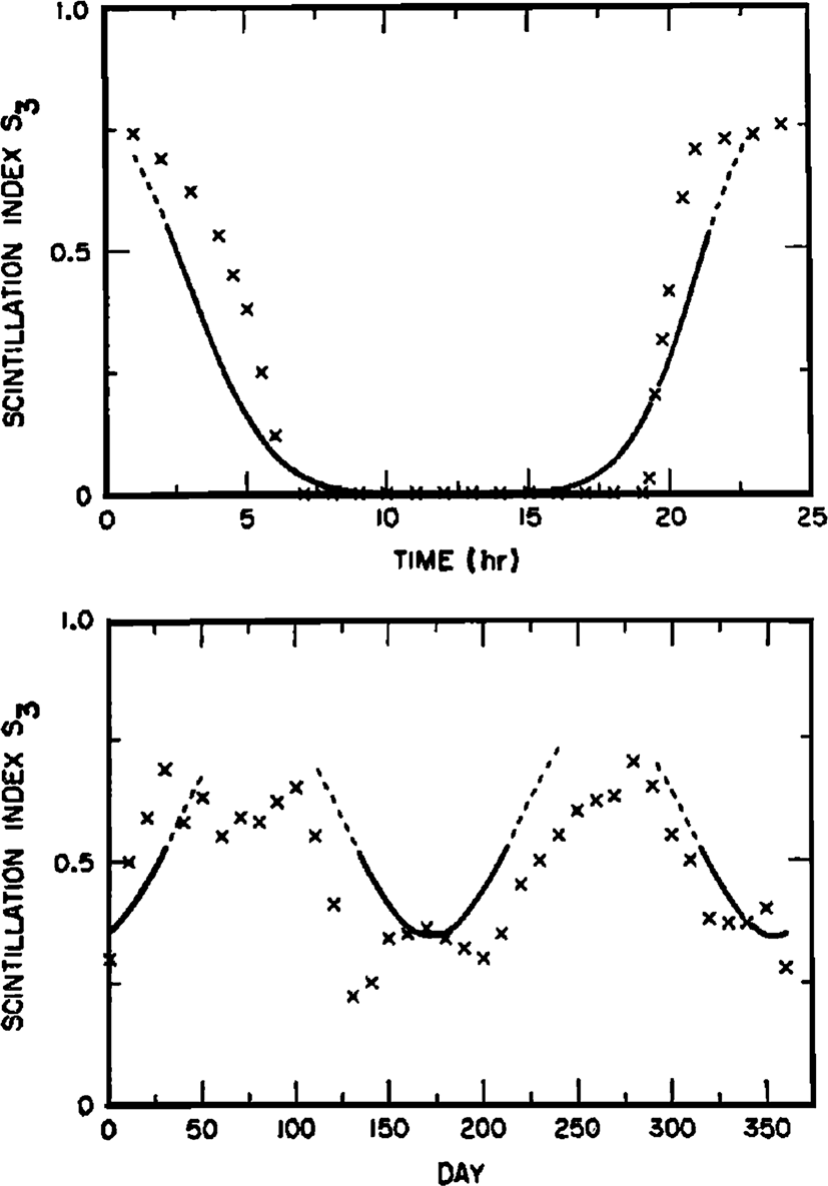
Comparison of model calculations with geostationary satellite observations from Ghana. The *top* is diurnal variation: frequency = 136 MHz, sunspot number = 107 and day number = 31. The *bottom* is seasonal variation: frequency = 136 MHz, sunspot number = 97, and time is 0200. The observations are shown as *discrete points* and the calculations as a *curve*. The *curve* is *solid* where the weak-scatter assumption is valid and *dashed* where it is questionable. Where it is invalid, no calculated results are given (after Fremouw and Rino 1973)

Figure 3 compares the model with geostationary satellite observations from Ghana. In this figure, the fits are reasonably close where the weak-scatter assumption holds. This model has a historical value, but it led to the foundation of a much more advanced model, WBMOD, which is discussed later.

#### Aarons Model

The Aarons model (1985) gave an understanding of equatorial scintillation outages and a means of dealing with them at specific geographic locations. Using 15-min peak-to-peak scintillation indices taken over 5 years, an empirical formula was developed to yield the average value of the scintillation index. It used observations made at Huancayo, Peru, on the magnetic equator from the LES 6 satellite transmitting at 254 MHz. The azimuth angle was 75°, and the average elevation angle was 45°. The data set has limitations. However, one limitation was that 22.00–24.00 LT observations were not available (the satellite beacon was turned off). This data set was used from ATS 3 at 137 MHz. The second limitation was the signal-to-noise ratio which resulted in a limiting value of approximately 16–19 dB excursion peak to peak. Other experiments near 250 MHz show much higher values. The output was given as mean decibels of fading peak to peak

28 $$SI({\text{dB}}) = 2^{(q + r)}$$

where

$$\begin{aligned} q & = FA + FB + ( - 1.5FA + 0.8FB)\, \cdot \,\cos \left[ {(\pi /12)(H - 0.2 - 0.25Kp)} \right], \\ r & = FC\left\{ {\cos \left[ {(\pi /6)(H + 3.3)} \right] - 0.4\cos \left[ {(\pi /4)(H + 1.5)} \right]} \right\}; \\ FA & = ( - 2.7 - 0.3FD)(S/100); \\ FB & = - 0.2 + FD + (0.1 - 0.1FD)Kp; \\ FC & = ( - 1.6 + 0.7FD)(S/100) + 0.1Kp; \\ FD & = \cos (2\pi /365)(D + 1.3) - 0.6\cos (4\pi /365)(D - 4). \\ \end{aligned}$$

Here, *D* is the day number, *H* is the local time in hours, *S* the solar flux at 10 cm given in solar flux units (an sfu = 10^−22^ m^−2^ Hz^−1^), and *Kp* is the planetary magnetic index. All the angles are in radians.

**Fig. 4:**
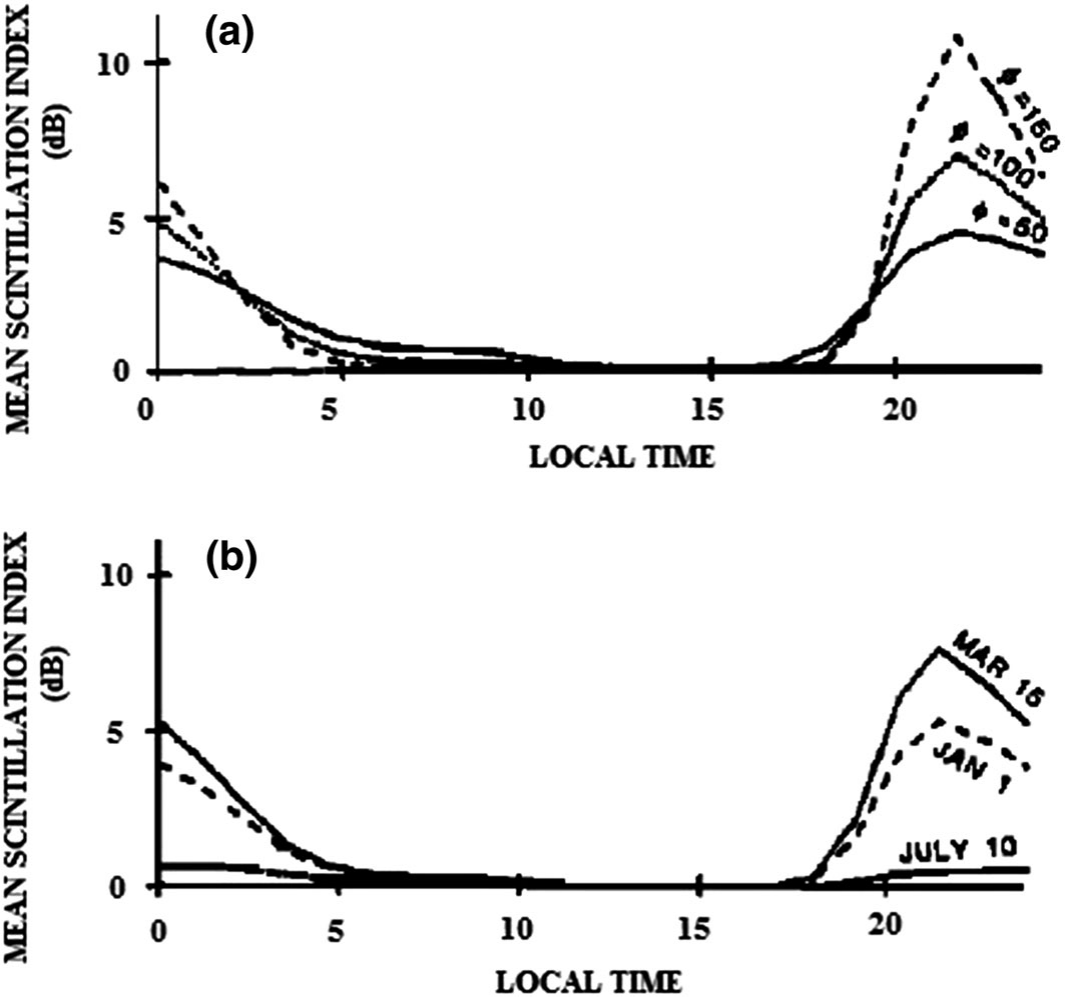
a. Mean scintillation index (in decibels peak-to-peak excursions) for February 15, with 10-cm solar flux of 50, 100 and 150 units; Kp = 2 (after Aarons, 1985). b. Scintillation index of the 3 days of the year, with solar flux of 100 and Kp = 2. The *solid line* is March 15, the *dashed line* is January 1, and *dotted line* is for July 10 (after Aarons 1985)

In Fig. 4a, the mean scintillation index is plotted on February 15, with three solar flux values of 50, 100 and 150, and with *Kp* = 2. Figure 4b shows the diurnal variation of the scintillation levels for three chosen days and for constant solar flux and *Kp*. The model has serious limitations in its application at other frequencies and other locations in the equatorial region.

#### Franke and Liu (1985) Model

This is an equatorial-latitude multi-frequency scintillation model. Analytical and numerical techniques have been used for modeling multi-frequency amplitude scintillation data observed at Ascension Island (equatorial region). The temporal coherence interval of multi-frequency amplitude scintillations observed at VHF, L band and C band has been studied by this model. The data used were a wide range of perturbation strengths corresponding to scintillation indices (*S*_4_) in the range 0.05–0.25 at C band (4 GHz). Franke and Liu (1985) modeled the multi-frequency behavior of the temporal coherence interval of amplitude scintillations due to two-component power law irregularities. They used both analytical and numerical models to solve the problem, and a phase screen has been used to model the propagation effects. They started by considering the multiplicative two-component model for the two-dimensional irregularities that was adopted by Franke et al. (1984). This two-dimensional model is reasonable because of the large elongation of equatorial irregularities along magnetic field lines (magnetic N–S alignment) and the nearly vertical propagation path for the experiment. For this model, the spectrum becomes

29 $$S_{\Delta N} (K) = \frac{{{\text{c}}_{\text{N}} }}{{\left( {K_{\text{o}}^{2} + K^{2} } \right)^{{{\text{P}}_{ 1} /2}} \left( {K_{\text{b}}^{2} + K^{2} } \right)^{{ ( {\text{P}}_{ 2} - {\text{P}}_{ 1} )/2}} }}$$

where *C*_*N*_ is a normalization constant, *K* ^2^ is *K*_*x*_^2^ + *K*_*z*_^2^, where *K*_*x*_ and *K*_*z*_ are the horizontal and vertical wave numbers, respectively. *K*_o_ is the outer scale wave number, and *K*_b_ the break scale wave number. It is assumed that *K*_b_ > *K*_o_. p_1_ and p_2_ are low-frequency and high-frequency power law indices. *C*_*N*_ can be expressed as

30 $$C_{N} = \frac{{\sigma_{N}^{2} }}{2\pi } \cdot \frac{{(K_{\text{b}}^{2} + K^{2} )}}{{\ln \left( {K_{\text{b}} + K_{ 0} } \right)}}$$

where σ_*Ν*_^2^ is the variance of the electron density fluctuations. Using expressions from Yeh and Liu (1982)

31 $$S_{\phi } (K_{X} ) = 2\pi \lambda^{2} r_{\text{e}}^{2} \sigma_{N}^{2} LS_{{\Delta {\text{N}}}} (K_{\text{X}} ,0)$$

where **λ** is the wavelength in meters, *r*_*e*_ is the classical electron radius, i.e., 2.82 × 10^−15 ^m, and *L* is the slab thickness of the irregularity regions in meters.

The spectrum of phase fluctuations in the phase screen can be written as

32 $$S_{\phi } (K_{X} ) = \frac{{C_{\phi } }}{{\left( {K_{\text{o}}^{2} + K_{\text{x}}^{2} } \right)\left( {K_{\text{b}}^{2} + K_{\text{x}}^{2} } \right)}}$$

where

$$C_{\phi } = \frac{{\sigma_{\phi }^{2} }}{\pi } \cdot K_{\text{o}} K_{\text{b}} (K_{\text{b}} + K_{\text{o}} )$$

The variance of the phase fluctuations in the screen is related to the electron density fluctuations as follows:

33 $$\sigma_{\phi }^{2} = \pi \lambda^{2} r_{\text{e}}^{2} \sigma_{N}^{2} L\frac{{K_{\text{b}} - K_{\text{o}} }}{{K_{\text{b}} K_{\text{o}} }} \cdot \frac{1}{{\ln (K_{\text{b}} /K_{\text{o}} )}}$$

For the weak scintillation case so that σ_ϕ_^2^ ≪ 1, the scintillation index *S*_4_ can be found (Yeh and Liu 1982)

34 $$\begin{aligned} S_{4}^{2} & = 4\int\limits_{ - \infty }^{\infty } {S_{\phi } (K_{X} )\sin^{2} \left( {\frac{{K_{\text{X}}^{2} z}}{2k}} \right){\text{d}}K_{\text{X}} } \\ & = 2\sigma_{\phi }^{2} \left\{ {1 - \frac{1}{{1 - \frac{\alpha }{\beta }}}} \right.[\cos (2\alpha^{2} ) \\ & - \sqrt 2 \cos \left( {2\alpha^{2} + \frac{\pi }{4}} \right)C(\sqrt 2 \alpha ) \\ & - \sqrt 2 \sin \left( {2\alpha^{2} + \frac{\pi }{4}} \right)S(\sqrt 2 \alpha )] \\ & - \frac{1}{{1 - \frac{\beta }{\alpha }}}[\cos (2\beta^{2} ) \\ & - \sqrt 2 \cos \left( {2\beta^{2} + \frac{\pi }{4}} \right)C(\sqrt 2 \beta ) \\ & - \sqrt 2 \sin \left( {2\beta^{2} + \frac{\pi }{4}} \right)S(\sqrt 2 \beta )] \\ \end{aligned}$$

where $\alpha = l_{\text{f}} ,\quad \beta = l_{\text{f}} K_{\text{b}} ,\quad l = \left( \frac{z}{2k} \right)^{1/2}$ and z is the distance from the phase screen to the receiver plane, and *C*(*x*) and *F*(*x*) are Fresnel integrals

35 $$S(x) = \left( {\frac{2}{\pi }} \right)^{1/2} \int\limits_{0}^{x} {\sin t^{2} {\text{d}}t}$$

36 $$C(x) = \left( {\frac{2}{\pi }} \right)^{1/2} \int\limits_{0}^{x} {\cos t^{2} {\text{d}}t}$$

If **α** = **β** and **α** ≪ 1, the model reduces to a single-component spectrum with *p* = 4 and outer scale size *L*o ≫ *ℓ*_*f*_. In this condition, the equation for *S*_4_ reduces to

37 $$S_{4}^{2} = 6.02\sigma_{\phi }^{2} (K_{\text{o}} l_{f} )^{3}$$

**Fig. 5:**
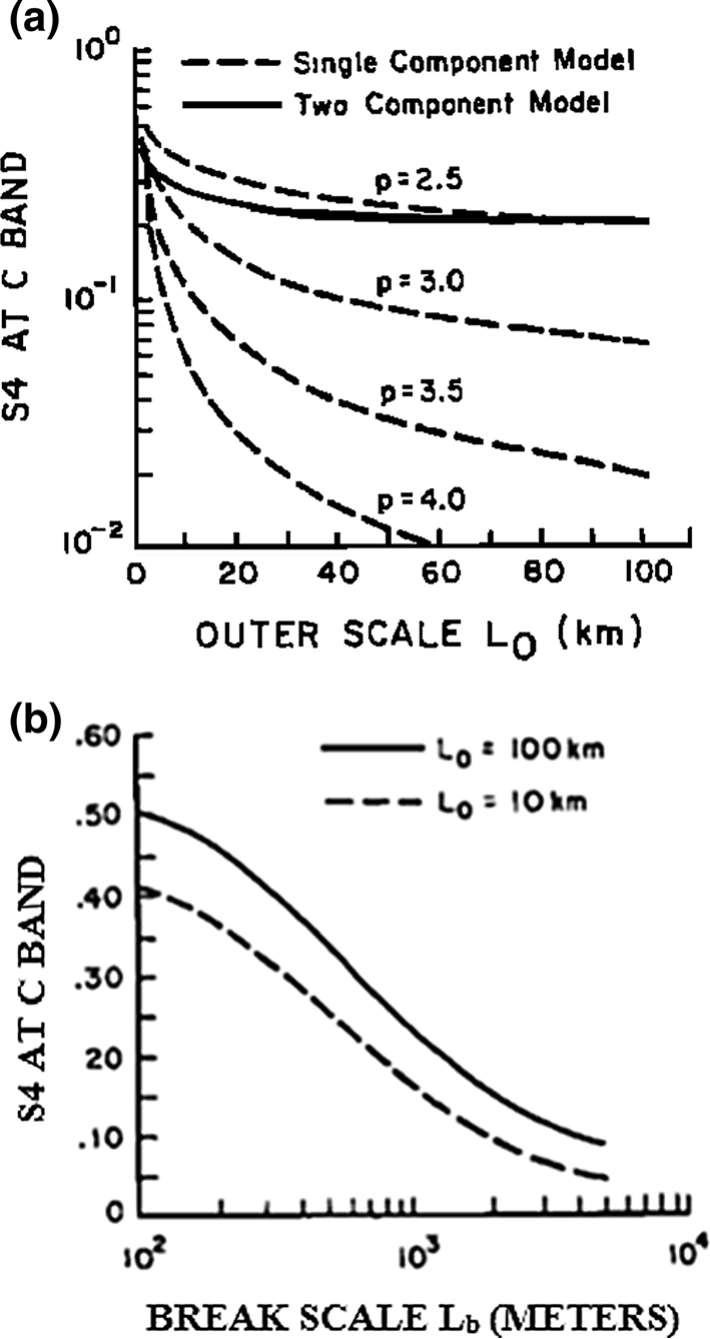
**a** Scintillation index at C band versus outer scale size *Lo*, in km (after Franke and Liu 1985). **b** Scintillation index at C band verses the break scale size in meters (after Franke and Liu 1985)

This is similar to Rino’s (1979) results for the single-component spectrum. Figure 5a shows the result of this model computation using Eq. (21). The parameters chosen are *f* = 3,945.5 MHz, σ_*Ν*_^2^ *L* = 1.08 × 10^29^ m^−5^ and *z* = 350 km. The break scale *L*_b_ is 750 m. The *S*_4_ index is plotted versus the outer scale size *Lo*. Also shown (dotted lines) are the results of model computations for single-component spectra with *p* = 2.5, 3.0, 3.5 and 4.0. In this model, the authors consider the case where the power law indices are given by pl = 2 and p2 = 4. These are two-dimensional power law indices; the corresponding one-dimensional power law indices are p1^(1)^ = 1 and p2^(1)^ = 3.

Figure 5b shows *S*_4C_ vs. L_b_ for outer scale sizes *L*_0_ = 10 and *L*_0_ = 100 km. The propagation distance z = 350 km. The dependence on the break scale is strong; to produce a scintillation index of ~0.2, the break scale is ~1 km for *L*_0_ = 10 km and ~700 m for *L*_0_ = 100 km.

In effect, the break scale assumes the role of the outer scale in the two-component model. If we define the coherence distance (d_I_) of the electron density perturbation as the spatial separation at which the normalized intensity covariance is 0.5, i.e., *C*_I_(*d*_I_) = 0.5 (Franke and Liu 1985), then it can be expressed in terms of the electron density perturbation as

38 $$d_{\text{I}} \simeq \left[ {\frac{{0.693K_{\text{b}} /K_{\text{o}} }}{{\pi \lambda^{2} r_{\text{e}}^{2} \sigma_{N}^{2} L(K_{\text{b}} ,K_{\text{o}} )}}} \right]^{1/2} \quad d_{\text{I}} \ll L_{\text{b}}$$

This was the result for the analytic model for the two-component spectrum. This model also demonstrates the consistency of the observational data with analytical and simulation results based on an irregularity spectrum. It shows that the coherence interval at VHF is a good indicator of the scintillation strength. Empirical formulas were derived based on the simulation results which relate the VHF coherence distance to the scintillation index at C band. These results were found useful for obtaining an approximate estimate of the scintillation strength at GHz frequencies based on the measurements of saturated VHF scintillations only. A simple inverse relationship was found to exist between the correlation interval of saturated scintillations at VHF and the perturbation strength as measured by the C band scintillation index.

#### Iyer et al. (2006) Model

The amplitude scintillation of 250-MHz signals from the geostationary satellite FLEETSAT (at 73° longitude E) was measured at the Indian magnetic equatorial station, Trivandrum, and at the anomaly crest station of Rajkot. The scintillation data recorded during the years 1987–1989 were used (Iyer et al. 2006).

**Fig. 6:**
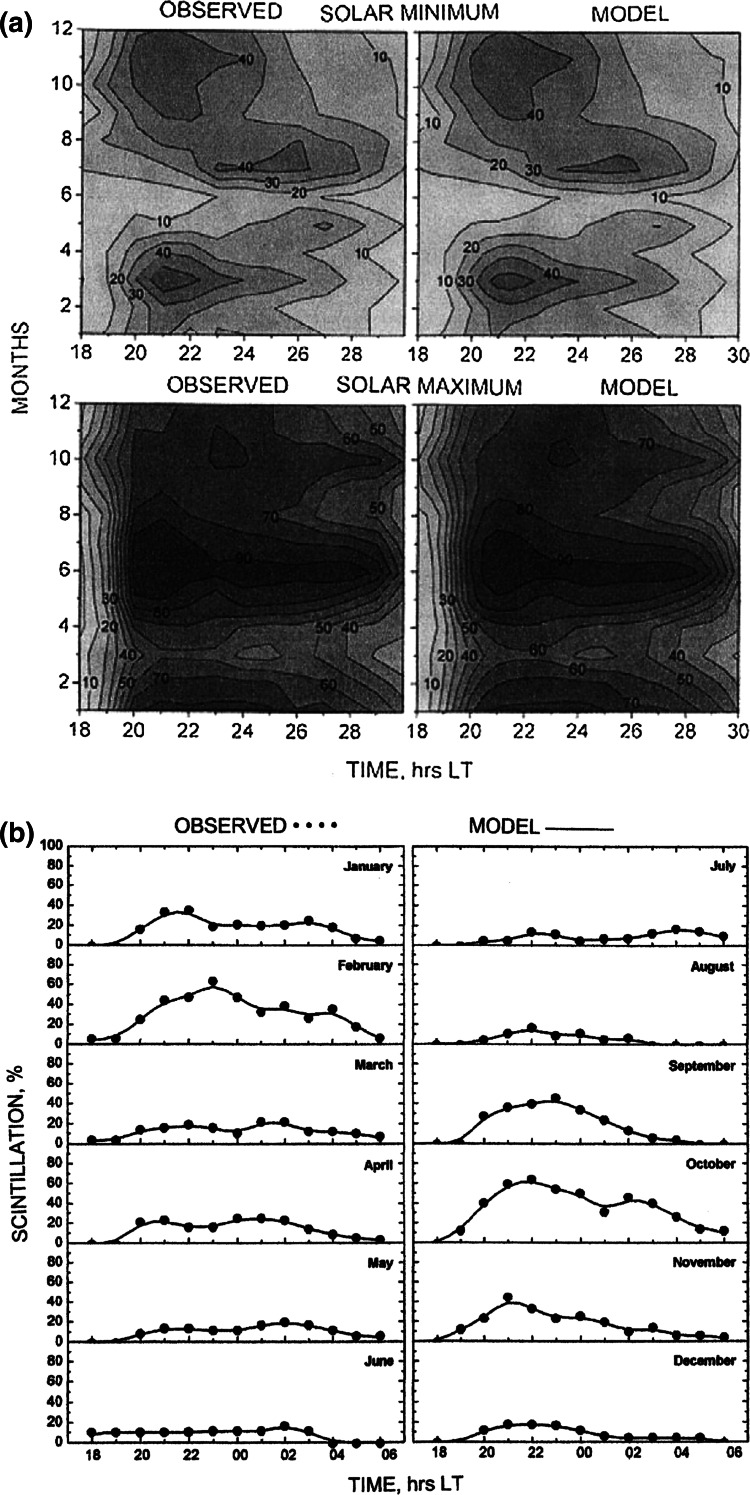
**a** Scintillation occurrence over Trivandrum for solar minimum (*upper panels*) and maximum (*lower panels*) (after Iyer et al. 2006). **b** Local time variation of scintillation occurrence over the anomaly crest station Rajkot for solar maximum (after Iyer et al. 2006)

The model takes into account seasonal, solar activity and latitudinal variations of the scintillation occurrence. Scintillation occurrence (SO, as a percentage), as functions of local time, latitude, season/day and solar flux value, is expressed as a simultaneous product of univariate normalized cubic B-splines as given below:

39 $$SO(t,d,F,\theta ) = \sum\limits_{i = 1}^{17} {\sum\limits_{j = 1}^{12} {\sum\limits_{k = 1}^{3} {\sum\limits_{l = 1}^{2} {a_{i,j,k,l} N_{i,4} (t)N_{j,2} (d)N_{k,2} (F)N_{l,2} (\theta )} } } }$$

where *t* is the local time, *d* is the day of the year, solar flux *F*, *a*_*i,j,k,l*_ are the monthly means of the scintillation occurrence percentage for each interval of local time and latitude θ expressed as a simultaneous product of univariate normalized cubic B-splines. The second subscript is the order of a cubic B-spline. The 17 local time nodes are distributed between 16.00 and 08.00 LT at one-hour intervals. The 12 seasonal nodes are placed at the 15th day of the month. In Fig. 6a, the modeled (right panels) and observed (left panels) scintillation occurrence in Trivandrum is compared for high and low solar activity. One may note a close agreement between observed and model values.

As evidenced by Fig. 6b, an excellent agreement has also been achieved for the anomaly station, Rajkot. In spite of the superb agreement between the model and observation, the usefulness of this kind of model in the prediction of scintillation levels is limited to the frequency and location for which it has been constructed.

#### 3D Ionospheric Plume Models

This model developed by Retterer (2010) is a three‐dimensional model for the plasma plumes caused by interchange instabilities in the low‐latitude ionosphere. It describes the structure and extent of the radio scintillation generated by turbulence around and within the plumes (Retterer 2010).

The model can predict the strength of radio scintillations as a function of time, latitude and longitude, given the drivers for the ionospheric structure (the plasma drift velocity and temperature) and the thermospheric parameters. Although the model cannot encompass a first‐principle description of phenomena on all the scale lengths relevant for the generation of scintillation, the necessary extrapolations are based on observations and physical principles. The phase screen formula is accurate only for weak scattering.

For power law density irregularity spectra, comparison with full-wave equation solutions (Dashen and Wang 1993) shows that the phase screen formula does describe the resulting amplitude scintillation accurately when the irregularities are weak. When the irregularities are stronger, however, the full-wave values of *S*_4_ saturate at a value near unity. Physically, this results because negative fluctuations in intensity cannot exceed the signal intensity in magnitude, nor can positive fluctuations exceed the average intensity for long. To derive the actual strength of scintillation, *S*_4_, the authors impose this saturation on the phase screen results, *S*_4ps_, using a simple analytical formula derived by visual inspection of numerical simulations results (Dashen and Wang 1993):

40 $$S_{ 4} = 1{-}{ \exp }\left( { - S_{{ 4 {\text{ps}} }} - S_{{ 4 {\text{ps}}}}^{2} } \right)$$

**Fig. 7:**
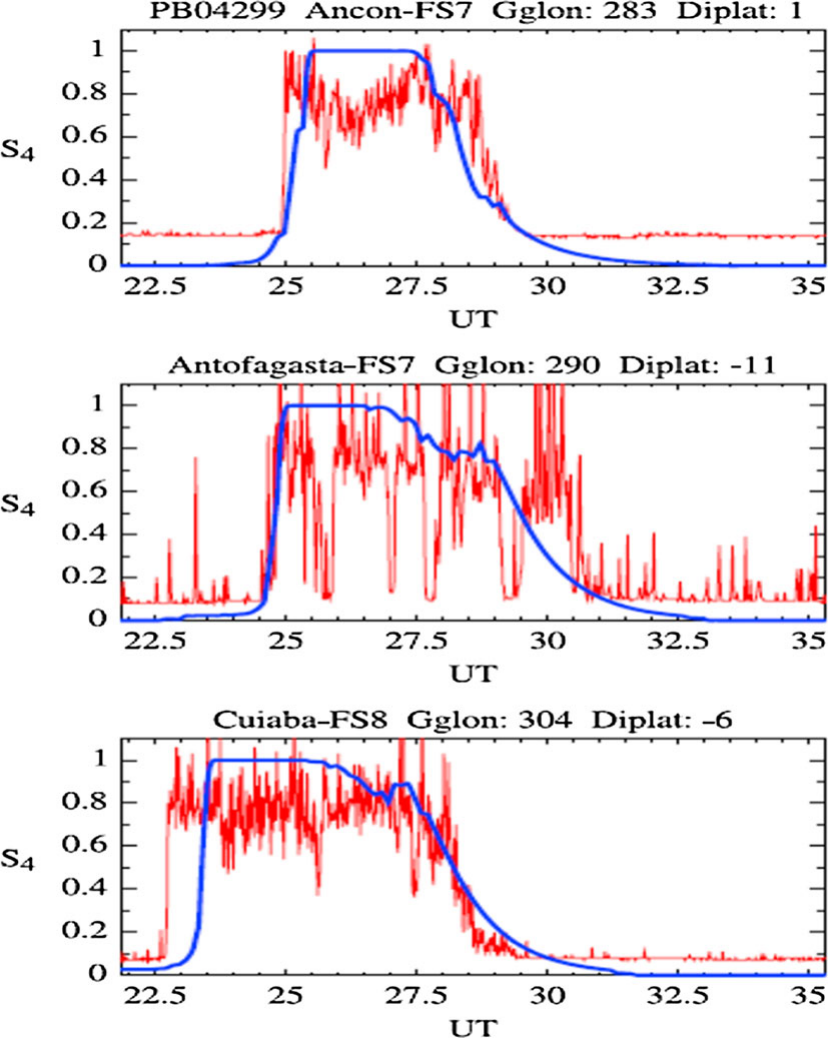
Comparison of predicted scintillation (*blue curves*) with UHF SCINDA (Scintillation Network Decision Aid) measurements (*red curves*) of amplitude scintillation at three South American stations (after Retterer 2010)

Figure 7 presents the *S*_4_ scintillation index reported for three stations, namely Ancon, Antofagasta and Cuiaba (their geomagnetic latitudes are given in Fig. 7) at 82 s sampling times using red curves, along with the scintillation index predicted by the model (blue line). At all three stations, the model does well in predicting the onset time and duration of the scintillations. This model provides an envelope within which the actual *S*_4_ varies.

### Global Climatological Models

#### WBMOD

The WBMOD (for WideBand MODel) ionospheric scintillation model was developed over the past two decades by NorthWest Research Associates (NWRA) with support from the US Government. This model can be used to calculate estimates of the severity of scintillation effects on a user-specified system and scenario (location, date, time, geophysical conditions). WBMOD consists of an ionosphere model, which provides the global distribution and synoptic behavior of the electron density irregularities that cause the scintillations, and a propagation model that calculates the effects that these irregularities will have on a given system (http://www.nwra.com/ionoscint/wbmod.html). The outputs that the model returns are the phase scintillation spectrum spectral index *p*, the spectral strength parameter (the spectral power at 1 Hz) *T*, the intensity scintillation index *S*_4_, and rms phase *σφ*.

**Fig. 8:**
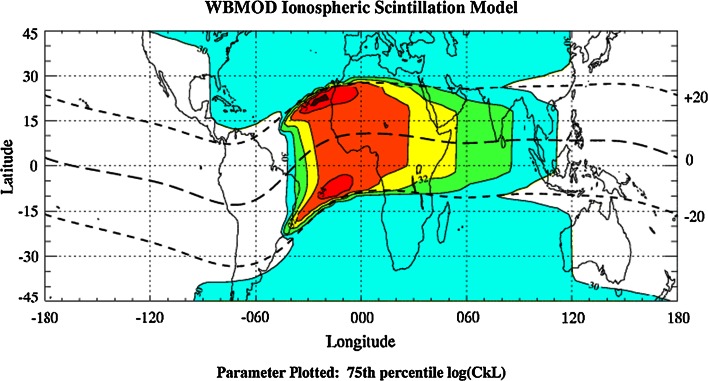
Contour map of log(*C*_*k*_ *L*) for 23.00 UT on March 21, 1994, and the sunspot number 150 (after www.nwra.com/ionoscint/wbmod.html)

The WBMOD consists of two parts: the electron density irregularities model and the propagation model. The electron density model was developed based on a large collection of scintillation observations taken during the Wideband, HiLat, and Polar Bear experiments and from the USAF Phillips Laboratory equatorial scintillation monitoring network. It provides information on the geometry, strength, orientation and motion of irregularities as a function of location (latitude, longitude), date, time of day, solar (sunspot number) and geomagnetic (planetary *K* index, *K*_*p*_) activity. The most important parameter returned by the model is the height-integrated irregularity strength *C*_*k*_ *L*, i.e., the product of the turbulence strength parameter *C*_*k*_ and the irregularity layer thickness *L.* An example of the contour plot of observed log(*C*_*k*_ *L*) is shown in Fig. 8. The highest values of log(*C*_*k*_ *L*) form just after local sunset on both sides of the magnetic equator (long dashed line). One can expect that these are the regions of strongest scintillation for the given conditions. The propagation model employed in WBMOD is the phase screen model as formulated by Rino (1979) and briefly described in Sect. 2.2.3. The phase spectrum is characterized by the power law with two-dimensional spectral index *p* and *T*—the phase spectral power at 1 Hz.

These are related to the properties of the electron density irregularities and geometry (Fremouw and Secan 1984):

41 $$p \approx q + 1$$

42 $$T = N\left( q \right)\lambda^{ 2} (C_{k} L){ \sec }\theta GV_{e}^{q}$$

where *q* is the one-dimensional spectral index of electron density fluctuations as measured in situ onboard a satellite, *λ* is the radio wavelength, *θ* is the propagation angle, *G*(*a, b,* *δ*) is a geometrical enhancement factor, and *V*_*e*_(***V***_*s*_, ***V***_*d*_ *, a, b,* *δ*) is the effective ray path scan velocity across contours of the plasma density; *N*(*q*) is a normalization factor. Other quantities are as follows: *a*—axial ratio along the magnetic field, *b*—axial ratio across the magnetic field, *δ—*orientation of sheet-like irregularities with respect to the *L*-shell, ***V***_*s*_—the line-of-sight scan velocity, ***V***_*d*_—large-scale drift velocity of irregularities.

Near the equator, WBMOD uses a simple model of drift velocity *V*_*d*_ varying diurnally, with eastward drifts reaching 100 m/s at 22.00 local time, westward drifts reaching 50 m/s at 10.00 L.T., and reversals taking place just before 16.00 and just after 04.00. It is assumed that, in the equatorial region, the axial ratio *b* is unity, so that irregularities are axially symmetric highly elongated rods with *a* = 30. As a measure of the phase scintillation, the phase variance is used, which is simply the integral of the phase spectrum *P*(*f*):

43 $$\sigma_{\phi }^{2} = \int\limits_{{f_{c} }}^{\infty } {P_{\phi } (f)} {\text{d}}f = 2\int\limits_{{f_{c} }}^{\infty } {\frac{{T{\text{d}}f}}{{(f_{0}^{2} /f^{2} )^{p/2} }}}$$

where *f*_0_ = *V*_*e*_/2*πr*_0_ and *r*_0_ is the outer scale. *f*_*c*_ is the lowest frequency admissible by the system, for instance, the phase detrending frequency. Usually, *f*_*c*_ ≫ *f*_0_ so that

44 $$\sigma_{\phi }^{2} \approx \frac{2T}{{(p - 1) f_{c}^{p - 1} }}$$

The intensity scintillation is measured using the scintillation index, defined as the normalized (by the mean squared) variance of the intensity:

45 $$S_{4}^{2} = \frac{{\left\langle {I^{\text{2}} } \right\rangle - \left\langle I \right\rangle^{2} }}{{\left\langle I \right\rangle^{2} }}$$

For weak intensity scintillation, the WBMOD uses following formula:

46 $$S_{4w}^{2} = \frac{M(q)}{N(q)}T\frac{F}{G}\frac{{Z^{q/2} }}{{V_{e}^{q} }}$$

where *F*(*q, a, b,* *δ*) is the Fresnel filter factor, *Z*(*λ*, *h*) is the Fresnel zone size, and *M*(*q*) is the normalization factor.

It is important to realize that the model is valid only for weak scintillation. If the scintillating signal obeys Rice statistics, then the following formula can be used to account for the saturation of the scintillation index *S*_4_:

47 $$S_{4}^{2} \approx 1 - \exp ( - S_{4w}^{2} )$$

where *S*_4*w*_ is the weak-scatter scintillation index.

**Fig. 9:**
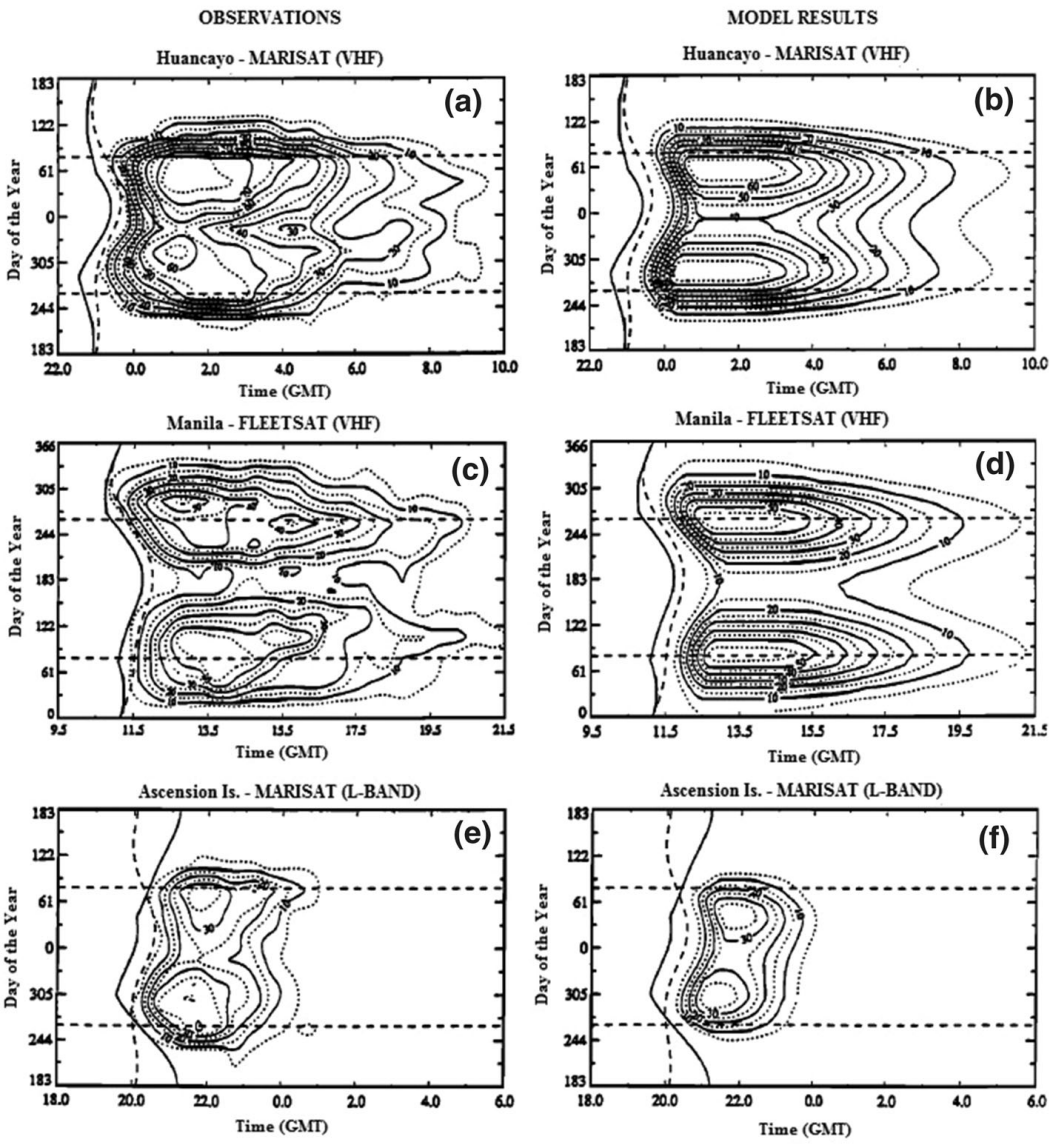
Variation of the percent occurrence of *S*_4_ > 0.5 as a function of UT and day of the year for indicated receiver location. *Left plots*—observations, *right plots*—model. Heavy *solid curves* indicate the time of *E* region sunset and heavy *dashed curves* the time of *F*2 region sunset. Note that Manilla is plotted with the June solstice at the center of the *y-*axis, while Huancayo and Ascension Island have the December solstice at the center (after Secan et al. 1995)

An improved WBMOD of equatorial scintillations can be found in Secan et al. (1995). Compared to the earlier model, the authors here use a more extended scintillation database. Thanks to that, it was possible to derive and use the probability distribution function of log(*C*_*k*_ *L*) instead of the average value of log(*C*_*k*_ *L*). This enables the use of the full scintillation statistics, which are needed to calculate the percentage of time that the scintillation exceeds a given level. It also permits calculation of the scintillation level at a user-specified percentile. In Fig. 9, the contours of the percent occurrence of *S*_4_ > 0.5 are plotted for the observed and modeled scintillation as a function of UT and day of the year for three selected locations in the equatorial region. One can see that the model is consistent with the observations. In particular, it adequately reproduces the longitudinal differences in the solstitial behavior of the scintillation activity between Manila, where the scintillation activity peaks during the June solstice, and Huancayo, where the highest level of scintillation is observed during the December solstice. WBMOD is a very popular model, and it is no wonder that there are a good number of papers dealing with its validation. We mention here the papers by Cervera et al. (2001) and Forte and Radicella (2005).

Cervera et al. (2001) used GPS scintillation data collected during 1998 and 1999 from two sites, one situated in the southern equatorial anomaly region and the other situated near the geomagnetic equator, in Southeast Asia. It has been found that at both the equatorial and anomaly sites, in 1998 when the solar activity was lower than in 1999, the modeled occurrence of scintillation stronger than *S*_4_ = 0.3 agreed with observations, although some differences were noted. However, in 1999 at the equatorial site, the predicted scintillation activity was much lower than that observed and this disagreement grows to be more serious for stronger scintillation. At the same time, in the anomaly region, the agreement of the model with observations was satisfactory. It has also been noted that scintillation activity predicted by WBMOD ceased approximately 2 h earlier than the observations showed. As indicated by Cervera et al. (2001), this can be of concern at VHF because at these frequencies the scintillation is strong and extends later into the night.

Forte and Radicella (2005) compared the WBMOD and GISM (see below) scintillation predictions with observations made in Tucuman (Argentina), at the crest of the equatorial anomaly. The authors highlight the patchy character of the equatorial scintillations which is not reflected in the models. Rather than that, the model predicts the average behavior of the scintillations as a function of time and position. That is why, for most of the time, the WBMOD fails to predict the scintillation on a given GPS link. Forte and Radicella (2005) underline the fact that the reaction of the GPS navigation system to scintillations depends on the receivers used in that different receivers might response differently to scintillations of a similar character (for instance, intensity and fading frequency).

#### GISM

The Global Ionospheric Scintillation Model (GISM) has been described by Béniguel and Buonomo (1999) and, in a slightly modified wording, by Béniguel (2002). The electron density is calculated by the model NeQuick developed by the University of Graz and ICTP Trieste (Radicella 2009). Inputs to this model are the solar flux number, the year, the day of the year and the local time. It provides the average electron density value for any point in the ionosphere (latitude, longitude, altitude). The magnetic parameters are computed based on a Schmidt quasi-normalized spherical harmonic model of the Earth’s magnetic field. These are the declination, the inclination, the vertical intensity and the components of the field.

The GISM uses the multiple phase screen technique (MPS) (Knepp 1983; Béniguel 2002; Béniguel et al. 2004; Gherm et al. 2005). The locations of transmitter and receiver are arbitrary. The radio link’s angle of incidence is arbitrary with respect to the ionosphere layers and to the magnetic field vector orientation. It can either cross the entire ionosphere or a small part of it. At each screen location along the line of sight, the parabolic equation (PE) is solved for estimating the complex amplitude. The ionospheric electron density at any point inside the medium, required for this calculation, is provided by the NeQuick model. Mean errors are related to the total electron content (TEC) value. The results are presented in the form of maps of scintillation index using geographic coordinates.

**Fig. 10:**
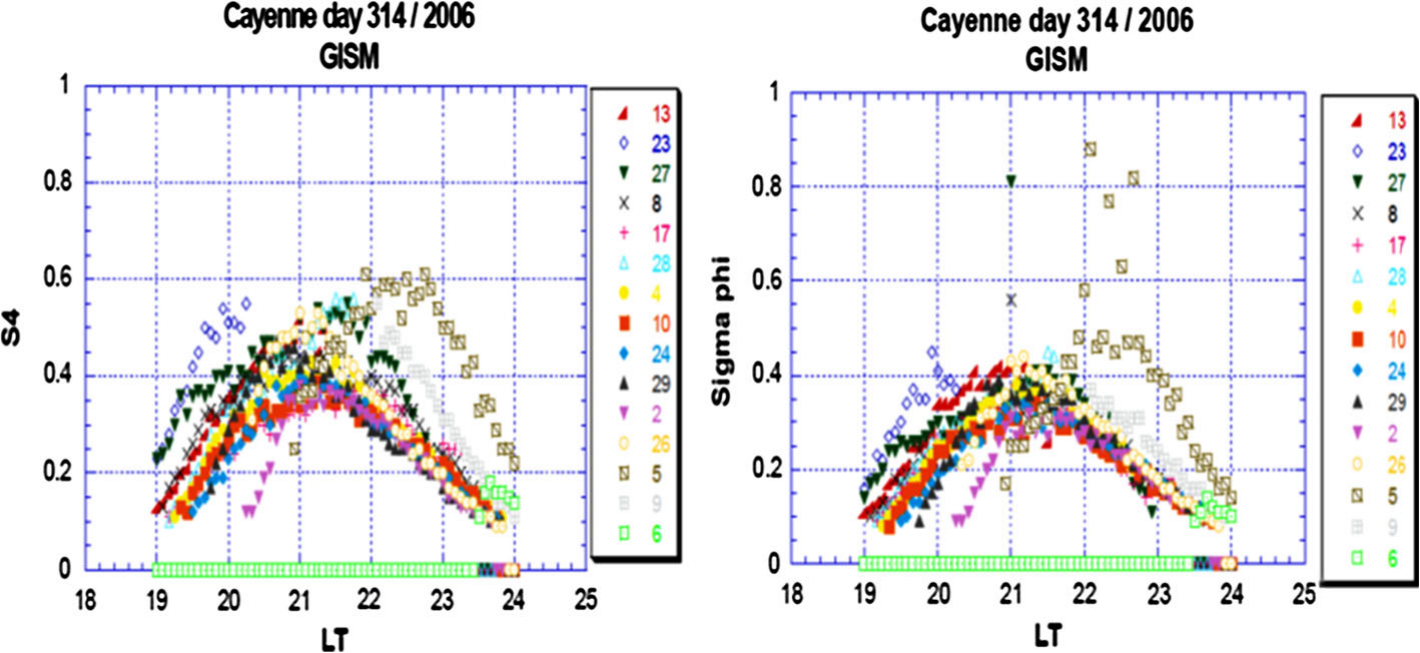
Intensity and phase scintillation indices on day 314, GPS week N° 377, obtained by modeling (after Béniguel 2011)

Forte and Radicella (2005) compared the GISM with observations collected over South America. As in the case of WBMOD, the patchy character of the low-latitude irregular structure is completely absent. GISM predicts the same behavior for scintillations at different local times, changing just the scintillation intensity, but not its morphology. It seems that WBMOD is more realistic as far as the reproduction of the diurnal scintillation variations is concerned. Figure 10 shows the plot of intensity and phase scintillation with local time at Cayenne, French Guiana, for 314 day of year 2006.

### Models Using Satellite in situ Data

#### Basu et al. (1976) Equatorial Scintillation Model

The model of Basu et al. (1976) is a morphological model of equatorial scintillations based on in situ irregularity measurements from OGO-6 satellite retarding potential analyzer (RPA) data. This instrument was used as a conventional RPA for 50 % of the time when ion temperature and ion composition were obtained. For the other half of the time, it was used as an irregularity detector. They further assumed that scintillation is weak and that a phase screen approximation as formulated by Rufenach (1975, 1976) is valid. The irregularity layer thickness was taken to be 200 km and its height to be 450 km. The outer scale of 20 km was chosen, and the axial ratio was considered to be greater than 5. Modeling was performed for vertical incidence.

**Fig. 11:**
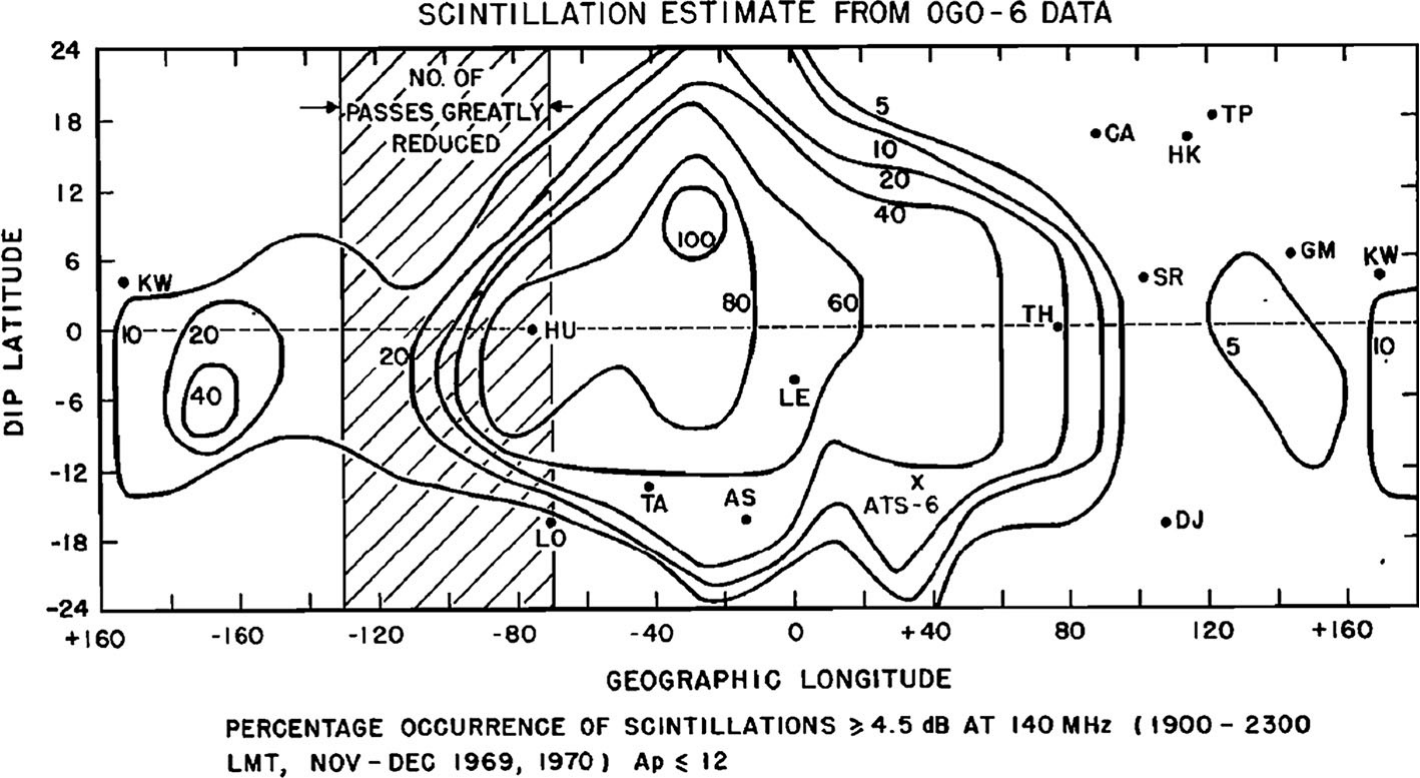
Contour plot of the occurrence of scintillations ≥4.5 dB at 140 MHz over the equatorial region (after Basu et al. 1976)

The percentage occurrence of scintillations estimated from the model is found to be consistent with observations of VHF scintillation at Ghana, Huancayo and Calcutta. The model demonstrates pronounced longitudinal variations in the scintillation activity with maximum values being in the African sector (Fig. 11).

#### High-Latitude Scintillation Models by Basu

Basu et al. (1981) used Atmospheric Explorer D (AE-D) data to model scintillations at high latitudes. Due to a limited availability of data, the model is suitable for northern winter under sunspot minimum conditions. Only rms plasma irregularity amplitude *σ*_*ΔN/N*_ computed from the satellite data over 3-s interval (approximately 20 km of path length perpendicular to the magnetic field). Values obtained every 8 s were used in the modeling. The first step in the modeling was to determine the behavior of *σ*_*ΔN/N*_ as a function of magnetic activity, magnetic latitude and magnetic local time.

The next step is to convert the plasma density morphology into the model of amplitude and phase scintillations. To accomplish this, Rino’s (1979) formulation of the phase screen theory of weak scintillation was used. The ambient electron density *N* and irregularity layer thickness *L* were derived from the Bent model of the ionosphere (Llewellyn and Bent 1973). It is assumed that the layer thickness is the same as the slab thickness of the ionosphere under similar geophysical conditions. To simplify the equations expressing the rms phase *σφ* and scintillation index *S*_4_ in terms of parameters characterizing the electron density irregularities and propagation geometry, it was assumed that the two-dimensional spectral index *p* = 3. These simplified equations are as follows:

48 $$\sigma_{\phi }^{2} \approx \frac{1}{{2^{5} \pi^{3} }}(r_{e} \lambda )^{2} (L\sec \theta )GC_{s} (v_{\text{eff}} \tau )^{2}$$

49 $$S_{4}^{2} \approx \frac{1}{2}(r_{e} \lambda )^{2} (L\sec \theta )GC_{s} \left( {\frac{\lambda z\sec \theta }{4\pi }} \right)F$$

where the turbulence strength parameter *C*_*s*_ = 2^3^ π 〈Δ*N* ^2^〉 (2π/*r*0), and τ is the phase detrend interval. The geometrical factors *G*, *F* and the effective scan velocity *v*_eff_ of the ray path across the electron density correlation ellipsoid depend on the anisotropy of the irregularities, the orientation of the geomagnetic field and the propagation angle. In the model, it was assumed that the scan velocity due to the satellite motion is much larger than scan velocity associated with the ionospheric drift. This 3 km/s scan velocity is in the magnetic N–S direction. Three kinds of irregularity anisotropy were considered. In the nighttime auroral oval, magnetic L-shell aligned E-W sheets were used, while in the daytime sub-auroral region, field-aligned rods were used.

#### WAM (Wernik et al. 2007) Model

The input data used in this Wernik et al. (2007) model are DE (Dynamic Explorer) 2 retarding potential analyzer (RPA) measurements of the ion density which, by overall charge neutrality, are equivalent to the electron density Ne. The satellite traversed a nearly polar orbit. The sampling frequency of the RPA was 64 Hz, corresponding to every 120 m along the satellite orbit. These measurements were grouped over 8-s (512 samples) segments. The data gaps not longer than three samples were filled using linear interpolation. Segments with longer gaps were rejected. Bad data, defined as that falling outside the interval ±4σ_*N*_ around the mean electron density for the segment, were corrected using linear interpolation. Only segments for which the invariant magnetic latitude was larger than 50° are considered.

**Fig. 12:**
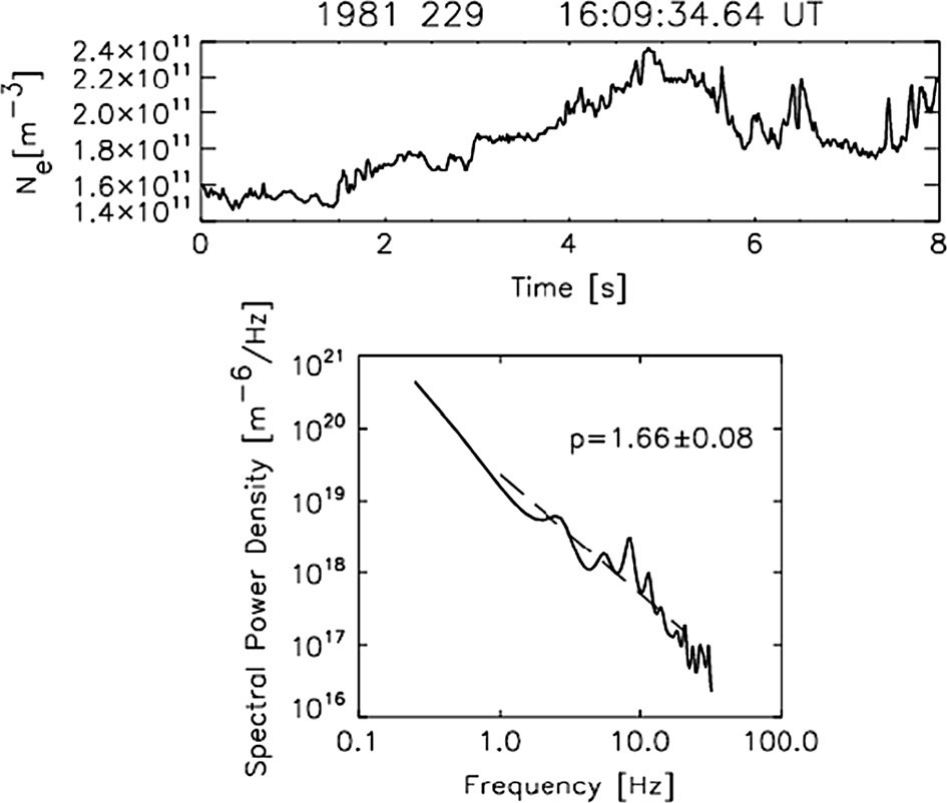
Example of 8-s-long data segment of ion density obtained by RPA on the DE 2 satellite and its maximum entropy spectrum. The *dotted line* f^−p^ dependence obtained by the least-squares fit over 1–20 Hz (after Wernik et al. 2007)

**Fig. 13:**
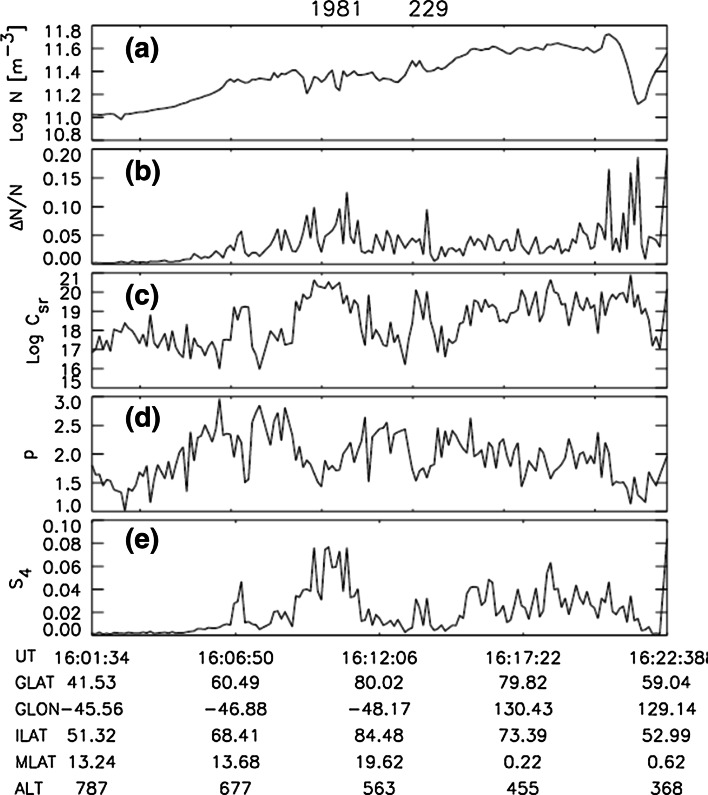
Modeling results for a single satellite path. **a** Segment of the measured density data. **b** Irregularity amplitude. **c** Turbulence strength parameter at the peak density height *C*_*sr*_. **d** Spectral index *p*. **e** Scintillation index *S*_4_ (after Wernik et al. 2007)

For each segment, Wernik et al. (2007) calculated the maximum entropy power spectrum (MEM) using 30 filter weights; altogether, they analyzed over 211,000 segments. Figure 12 presents an arbitrarily chosen data segment and its spectrum, and Fig. 13 presents an example of calculations for a single DE 2 satellite path. Figure 13a shows the log of the electron density averaged over data segments 8 s long, while Fig. 13b shows the irregularity amplitude. In Fig. 13c is plotted the log of turbulence strength parameter *C*_sr_ at the peak height. In Fig. 13d, the spectral index *p* is given, and in Fig. 13e, we show the scintillation index *S*_4_ at the signal frequency of 1.2 GHz. The high signal frequency was chosen to ensure that the scintillation is weak enough to comply with the weak-scatter assumption. This database is used to derive various statistically significant relationships and maps.

The model is limited by the data used in its construction. Since scintillation is strongly controlled by solar activity (Wernik et al. 2007) and since DE 2 was operating during a period of moderate solar activity, the model is expected to be valid only when the sunspot number is within the range 80–140. Another limitation is the assumption that the irregularities traversed by the probe are isotropic. This assumption leads to an overestimate of the turbulence strength parameter and consequently overestimated scintillation index. At the dip angle 60°, the error in *S*_4_ might be as large as 25 % for highly anisotropic irregularities, but decreases as the geomagnetic latitude increases. A serious limitation is imposed by the use of the International Reference Ionosphere (IRI) model, which often fails to give reasonable high-latitude F-region electron density profiles, so that important parameters such as the peak density, peak height and irregularity layer thickness might be erroneously estimated. Another source of the disagreement between the modeled S4 and the observations is an inaccurate model of irregularity anisotropy.

## Summary and Conclusions

In this paper, we have presented a general overview of models of ionospheric scintillations for high and equatorial latitudes. Trans-ionospheric communication of radio waves from transmitter to user is affected by the ionosphere which is highly variable and dynamic in time and space. Scintillation is the term given to irregular amplitude and phase fluctuations of the received signals and related to the electron density irregularities in the ionosphere. Key sources of ionospheric irregularities are plasma instabilities. Every model of ionospheric scintillations is based on the theory of radio wave propagation in random media.

Emphasizing the full structure of the complex signal is significant because it has been directly measured with a number of satellites having onboard multi-frequency phase coherent beacons. The small-scale irregularities in the F layer of the ionosphere produce amplitude fluctuations which can be a problem to navigation and communication systems in the very high or ultra-high frequency (VHF-UHF) ranges. Irregularity regions do exist at high latitude whose lower boundary at midnight is approximately 57° invariant latitude. At ±15° latitude on either side of the geomagnetic equator, ionospheric irregularities produce strong scintillations in the VHF range, particularly during post-sunset to pre-midnight hours.

The first empirical model of scintillations proposed by Fremouw and Rino (1973) could estimate the scintillation index *S*_4_ on VHF/UHF, under weak-scatter conditions. However, the weak-scatter condition is often exceeded near the equatorial anomaly and in auroral regions. This model led to the foundation of a more advanced model WBMOD. Aarons (1985) developed an analytic model using 15-min peak-to-peak scintillation indices (not *S*_4_) taken over 5 years at Huancayo, Peru, from LES 6 satellite signals transmitted at 254 MHz. Franke and Liu (1985) proposed the modeling of equatorial multi-frequency scintillations. Analytical and numerical techniques have been used to model multi-frequency amplitude scintillation data recorded in the equatorial region at Ascension Island. Later on came the model by Iyer et al. (2006). They used a cubic B-spline technique to develop an empirical model of magnetic quiet time scintillation occurrence at Indian equatorial and low latitudes. A 250-MHz signal from the FLEETSAT satellite was measured for 2 years at Trivandrum, near the magnetic equator, and at Rajkot at the crest of the equatorial anomaly.

To describe the structure and extent of the radio scintillation generated by turbulence around and within the equatorial plumes, a physical model was developed by Retterer (2010). The first climatological model WBMOD was developed by NorthWest Research Associates in which the user can specify his/her operating scenario. As an output, the model returns the phase scintillation spectral index *p*, the spectral strength parameter *T*, *S*_4_, and rms phase σ_ϕ_. Another global ionospheric scintillation model, GISM, has been described by Béniguel and Buonomo (1999). The model consists of two parts; these are the NeQuick model and the scintillation model based on a multiple phase screen algorithm, and a second part which needs statistical information about the irregularities as an input. The algorithm is used to calculate the scintillation index at the receiver.

Basu and his group used in situ satellite data in scintillation modeling for the first time in 1976. They assumed a 3D power law irregularity spectrum with a constant spectral index of 4. They prepared another high-latitude scintillation model (1988) using Atmospheric Explorer D data. Due to the limited availability of data, the model was only suitable for northern winter under sunspot minimum conditions. Wernik et al. (2007) used Dynamics Explorer B data to estimate the irregularity spectral index and turbulence strength parameter, the factors that are required to calculate the scintillation index (Rino 1979). Their approach has been extended by Liu et al. (2012) by introducing the finite outer scale.

This paper reviews many analytical, empirical and climatological models that place emphasis on the irregularities directly embedded in the background ionosphere. We did not consider the more critical review of the models since many authors (e.g., Forte and Radicella 2005; Strangeways et al. 2014) have already tried to make a direct numerical comparison between scintillation results achieved by various different models. In general, the ionospheric scintillation models discussed in this paper reproduce well the global morphology of the ionospheric scintillations, but they often show a lack of precision in the detailed resolution for short time periods (e.g., geophysical case studies). Strangeways et al. (2014) have presented a significant numerical comparison of four different models. They have demonstrated through curve comparison that different methods give approximately the same variation with the parameters (outer scale, elevation angle, power law of irregularity spatial spectrum and normalized variance of electron density irregularities) for weak scintillation conditions. There are few ionospheric scintillation models applicable to the irregularities in realistic larger structures such as equatorial bubbles at equatorial latitudes (e.g., Zernov et al. 2009) and polar patches at high latitudes (e.g., Maurits et al. 2008). Such modeling could be possible by using the hybrid scintillation propagation model (HSPM) method discussed by Gherm et al. (2005). They proposed a propagation model for trans-ionospheric fluctuating paths of propagation which is valid for strong scintillations and leads to a software trans-ionospheric channel simulator. The hybrid method, a combination of the complex phase method and the random screen technique, uses the extended Rytov approximation (the complex phase method). The HPSM technique was found to be capable of producing statistical characteristics and simulating time realizations of the field (including in the regime of strong amplitude fluctuations).
